# Spatial Segregation of BMP/Smad Signaling Affects Osteoblast Differentiation in C2C12 Cells

**DOI:** 10.1371/journal.pone.0025163

**Published:** 2011-10-05

**Authors:** Eva Heining, Raghu Bhushan, Pia Paarmann, Yoav I. Henis, Petra Knaus

**Affiliations:** 1 Institute for Chemistry and Biochemistry, Freie Universitaet Berlin, Berlin, Germany; 2 Department of Neurobiology, George S. Wise Faculty of Life Sciences, Tel Aviv University, Tel Aviv, Israel; University of Birmingham, United Kingdom

## Abstract

**Background:**

Bone morphogenetic proteins (BMPs) are involved in a plethora of cellular processes in embryonic development and adult tissue homeostasis. Signaling specificity is achieved by dynamic processes involving BMP receptor oligomerization and endocytosis. This allows for spatiotemporal control of Smad dependent and non-Smad pathways. In this study, we investigate the spatiotemporal regulation within the BMP-induced Smad transcriptional pathway.

**Methodology/Principal Findings:**

Here we discriminate between Smad signaling events that are dynamin-dependent (i.e., require an intact endocytic pathway) and dynamin-independent. Inhibition of dynamin-dependent endocytosis in fluorescence microscopy and fractionation studies revealed a delay in Smad1/5/8 phosphorylation and nuclear translocation after BMP-2 stimulation of C2C12 cells. Using whole genome microarray and qPCR analysis, we identified two classes of BMP-2 induced genes that are differentially affected by inhibition of endocytosis. Thus, BMP-2 induced gene expression of *Id1*, *Id3*, *Dlx2* and *Hey1* is endocytosis-dependent, whereas BMP-2 induced expression of *Id2*, *Dlx3*, *Zbtb2* and *Krt16* is endocytosis-independent. Furthermore, we demonstrate that short term inhibition of endocytosis interferes with osteoblast differentiation as measured by alkaline phosphatase (ALP) production and qPCR analysis of osteoblast marker gene expression.

**Conclusions/Significance:**

Our study demonstrates that dynamin-dependent endocytosis is crucial for the concise spatial activation of the BMP-2 induced signaling cascade. Inhibition of endocytic processes during BMP-2 stimulation leads to altered Smad1/5/8 signaling kinetics and results in differential target gene expression. We show that interfering with the BMP-2 induced transcriptional network by endocytosis inhibition results in an attenuation of osteoblast differentiation. This implies that selective sensitivity of gene expression to endocytosis provides an additional mechanism for the cell to respond to BMP in a context specific manner. Moreover, we suggest a novel Smad dependent signal cascade induced by BMP-2, which does not require endocytosis.

## Introduction

Bone morphogenetic proteins (BMPs) are members of the transforming growth factor-β (TGF-β) superfamily and elicit important roles in proliferation, embryonic development, differentiation and tissue regeneration [1], [2]. Urist and co-workers first identified BMPs as potent inducers of ectopic bone formation [3], [4]. Accordingly, these cytokines were shown to posses the potential to convert mesenchymal cells into osteoblasts or chondroblasts [5].

BMPs signal *via* two transmembrane serine/threonine kinase receptors, the BMP receptors type I (BMPRIa, BMPRIb, Alk2) and the BMP receptor type II (BMPRII, ActRII and ActRIIB). BMP receptors form homomeric and heteromeric complexes that exist in distinct membrane areas and are differently modulated by their ligands. BMP-2 binds to preformed heterocomplexes (PFCs) of BMPRI and BMPRII, initiating Smad-dependent signaling. In contrast, ligand binding to the high affinity receptor BMPRI induces the formation of heteromeric BMP-induced signaling complexes (BISCs), which regulate non-Smad signaling. Signaling *via* BISCs activates the mitogen-activated protein kinase (MAPK) signaling cascade, leading to induction of alkaline phosphatase (ALP) expression [6]. The Smad pathway is initiated by phosphorylation of regulatory Smads (Smad1/5/8), which subsequently associate with the common mediator Smad (Smad4), translocate into the nucleus, and regulate transcription of specific BMP target genes by recruiting additional activators or repressors [7].

Diseases ranging from skeletal diseases, vascular diseases, tissue dystrophies to cancer are caused by malfunctions of BMP signaling pathways. This highlights the importance of fine-tuning the BMP signaling responses. Extracellular antagonists such as Noggin control binding of the ligand to the receptor complexes [8]. Co-receptors like Ror2 and DRAGON or cytoplasmic co-regulators like inhibitory Smad7 affect the signal transduction into the nucleus and thereby modulate the signal propagation, resulting in transcriptional activation or repression [2].

Endocytosis of transmembrane receptors is an important mechanism to control receptor availability at the cell surface or to induce attenuation of signal transduction by sequestering receptors from modulators [9]. A well-described mechanism for cellular uptake is clathrin-mediated endocytosis. Thereby, clathrin-coated pits pinch off the membrane in a dynamin-dependent manner. Internalized receptors are either sorted for recycling to the plasma membrane, are degraded in lysosomes or can use the endosome as a signaling platform, where downstream components are presented for further activation. Another mode of internalization is uptake *via* caveolae-mediated endocytosis; this non-clathrin endocytic pathway uses membrane invaginations containing the membrane protein caveolin, and also requires dynamin for the pinching off of caveolar vesicles [10].

BMP receptors can enter the cell by different endocytic routes. Different intracellular cascades are initiated dependent on receptor localization in distinct compartments of the plasma membrane and their oligomerization mode [11]. Phosphorylation of Smad1/5/8 by BMPRI is induced at the plasma membrane, but the continuation of the Smad1/5/8 dependent pathway which results in transcriptional activation of specific target genes was shown to rely on clathrin-mediated endocytosis, while the Smad-independent pathway required both clathrin- and caveolae-mediated endocytosis to exert transcriptional activity [11].

In this study, we further investigated the dependence of the transcriptional regulation in BMP signaling on endocytosis and the consequences of endocytosis inhibition on osteoblast differentiation of C2C12 cells. By the use of dynasore, an inhibitor of dynamin-dependent endocytosis [12], we show that the kinetics of BMP-2 induced Smad1/5/8 phosphorylation and nuclear translocation are delayed and Smad1/5/8 phosphorylation is reduced. Although Smad1/5/8 phosphorylation and nuclear translocation still occur, the delay in the initial kinetics results in down-regulation of the BMP responsive element (BRE) reportergene activity and osteoblast differentiation. To provide more insight on Smad-dependent transcriptional activation, we performed a whole genome microarray analysis and identified two classes of BMP-2 induced genes, which display differential sensitivity to inhibition of dynamin-dependent endocytosis. Thereby, we could cluster genes into endocytosis dependent and endocytosis independent groups. Our data highlight the importance of endocytosis for transmission of an extracellular BMP-2 signal into the nucleus to activate a transcriptional gene network involved in osteoblast differentiation. Additionally, we suggest a novel Smad signal transduction cascade induced by BMP-2, which does not depend on endocytosis.

## Results

### The kinetics of Smad1/5/8 phosphorylation is influenced by dynamin-dependent endocytosis

BMP signaling is regulated by receptor oligomerization and membrane localization and its subsequent intracellular signal propagation is controlled by different endocytic routes [6], [11]. To gain further insights into the endocytic regulation of BMP signaling pathways, we used the small molecule inhibitor dynasore, which specifically interferes with dynamin-dependent endocytosis by reversibly blocking the GTPase activity of dynamin. Dynamin is crucial in vesicle fission prior to the release of vesicles from the plasma membrane [13]. Macia and colleagues showed that the inhibitor dynasore does not abolish vesicle formation but pinching off the membrane invaginations is blocked [12].

**Figure 1:**
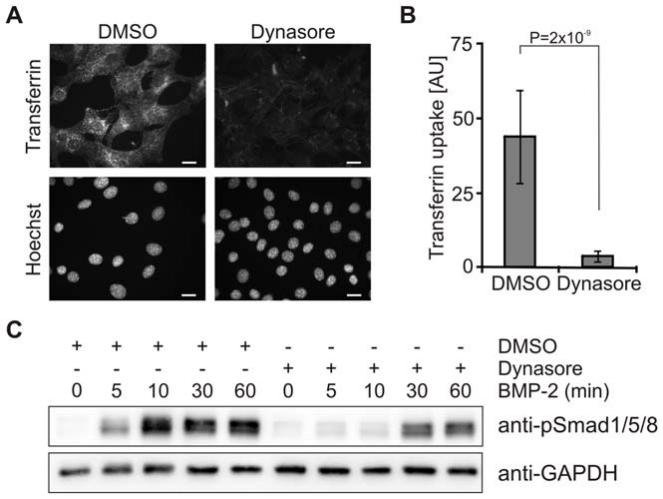
Phosphorylation of Smad1/5/8 is delayed by inhibition of dynamin-dependent endocytosis. (**A**) Serum starved C2C12 cells were treated for 2 h with 40 µM dynasore or 0.05% DMSO and incubated for 15 min at 37°C with Alexa594-transferrin in the presence of dynasore and DMSO. Cells were fixed and transferrin uptake was monitored by fluorescence microscopy. Lower panels show Hoechst staining of the respective cells. Bar, 10 µm. (**B**) Quantification of transferrin uptake shown in (A). The histogram depicts the amount of internalized transferrin, derived from the total fluorescence signal of Alexa594-transferrin within the cell boundaries. The results are mean ± s.d. of at least 400 cells. AU, arbitrary units. (**C**) Serum-starved C2C12 cells were treated for 2 h with 40 µM dynasore or 0.05% DSMO prior to stimulation with 10 nM BMP-2 for the indicated time periods in medium containing dynasore or DMSO. Samples were processed for immunoblotting with anti-phospho-specific Smad1/5/8 antibody (anti-pSmad1/5/8) or GAPDH antibody (anti-GAPDH) as loading control. The Western Blot shown is representative of three independent experiments.

To test the efficiency of dynasore ability to inhibit endocytosis in our system, C2C12 cells were subjected to fluorescent transferrin uptake to monitor clathrin-mediated endocytosis [14]. C2C12 cells incubated with Alexa594-transferrin or DMSO as control showed intracellular transferrin staining after 15 min (Figure 1A, left panels). In contrast, transferrin uptake was potently inhibited by treating the cells for 2 h with 40 µM dynasore (Figure 1A, right panels). The quantification of this experiment underlines the potential of dynasore to efficiently inhibit endocytosis in C2C12 cells (Figure 1B). In all subsequent assays transferrin uptake was measured in parallel to validate the treatment conditions with dynasore (data not shown).

We have previously shown that Smad1/5/8 phosphorylation is induced at the plasma membrane and it is not affected by treating cells with specific inhibitors for caveolae- or clathrin-dependent endocytosis [11]. To extend these findings, we now analyzed the effects of dynasore on Smad1/5/8 phosphorylation in a time-dependent manner (Figure 1C). C2C12 cells pre-treated with dynasore were stimulated for varying periods with BMP-2, followed by Western blotting with an antibody to C-terminally phosphorylated Smad1/5/8 (pSmad1/5/8). In control cells, phosphorylation of Smad1/5/8 was rapidly induced by BMP-2 after 5 min, peaked at 10 min, and stayed at sustained levels for up to 60 min (Figure 1C, lanes 1–5). The Smad1/5/8 phosphorylation kinetics was delayed and the levels were reduced in cells treated with dynasore. In the presence of dynasore, phosphorylation of Smad1/5/8 was much lower after 5 min to 10 min and became clearly apparent after 30 min to 60 min (Figure 1C, lanes 6–10). This result confirms that phosphorylation of Smad1/5/8 can occur at the plasma membrane prior to detaching of endocytic vesicles; yet, it shows that full activation of Smad1/5/8 is reliant on functional membrane dynamics.

### The nuclear translocation kinetics of Smad1/5/8 is regulated by dynamin-dependent endocytosis

**Figure 2:**
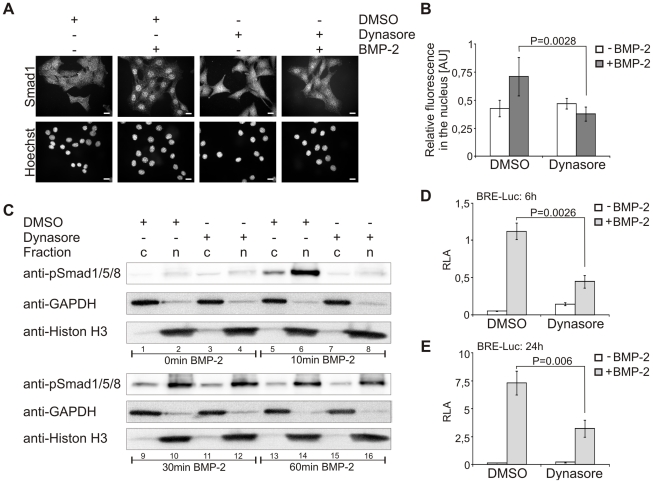
Smad1/5/8 nuclear translocation is delayed and transcriptional activity is reduced by inhibition of dynamin-dependent endocytosis. (**A**) Serum-starved C2C12 cells were treated for 30 min with 40 µM dynasore or 0.05% DMSO prior to stimulation with 10 nM BMP-2 for 30 min in medium containing dynasore or DMSO. After fixation, endogenous Smad1 was stained using a specific antibody, nuclei were stained by Hoechst dye, and cells were analyzed by fluorescence microscopy. The panels shown are representative of two independent experiments. Bar, 10 µm. (**B**) Quantification of the experiment shown in (A). Relative fluorescence intensity of nuclear to cytoplasmic Smad1 staining is depicted in the histogram. The results are mean ± s.d. of at least 100 cells. AU, arbitrary units. (**C**) Serum-starved C2C12 cells were treated for 2 h with 40 µM dynasore or 0.05% DSMO prior to stimulation with 10 nM BMP-2 for the indicated time periods in medium containing dynasore or DMSO. Samples were subjected to cytoplasmic-nuclear fractionation and processed for immunoblotting with anti-phospho-specific Smad1/5/8 antibody (anti-pSmad1/5/8). To control fractionation, samples were analyzed using anti-GAPDH or anti-Histon H3 antibodies. The Western Blot is representative of two independent experiments. c, cytosol; n, nucleus. (**D,E**) C2C12 cells were co-transfected with BRE-Luc and RL-TK. Cells were serum-starved and treated with 40 µM dynasore or 0.05% DMSO for 1 h prior to stimulation with 3 nM BMP-2 for 6 h (D) or 24 h (E) in medium containing dynasore or DMSO. Relative luciferase activity (RLA) of BRE-driven luciferase compared to constitutive expression of RL-TK is shown. Results are mean ± s.d. of triplicate measurements, representative of three independent experiments.

After phosphorylation, Smad proteins translocate into the nucleus to function as transcriptional co-regulators [7]. To further analyze the consequences of the altered Smad1/5/8 phosphorylation kinetics on the propagation of BMP signaling, we examined the effect of inhibition of dynamin-dependent endocytosis on Smad nuclear translocation. C2C12 cells were seeded on glass coverslips and treated with dynasore prior to stimulation with BMP-2 for 30 min. The subcellular localization of endogenous Smad1 was analyzed by fluorescence microscopy. BMP-2 induced strong nuclear accumulation of Smad1, which was significantly decreased in the presence of dynasore (Figure 2A,B). To confirm these results we performed cell fractionation studies. C2C12 cells were pre-treated with dynasore prior to stimulation with BMP-2 for the indicated time periods; they were then subjected to cytoplasmic-nuclear fractionation (Figure 2C). In control cells, phosphorylation of Smad1/5/8 was detectable in the cytosol and nucleus, with a strong peak in the nucleus after 10 min of BMP-2 administration (Figure 2C, lanes 5 and 6). Intense signals for nuclear pSmad1/5/8 were detected also after 60 min of BMP-2 stimulation, whereas the amount of pSmad1/5/8 in the cytosolic fraction decreased from 10 min to 60 min (Figure 2C, lanes 5, 6, 9, 10, 13, 14). As demonstrated in Figure 1C, dynasore delayed the initial phosphorylation kinetics of Smad1/5/8 (Figure 2C, lanes 5 and 7). Moreover, cells treated with dynasore and stimulated for 10 min with BMP-2 did not display phosphorylated Smad1/5/8 in the nuclear fraction (Figure 2C, lanes 6 and 8). Interestingly, phosphorylation of Smad1/5/8 in dynasore-treated cells was detectable in the nucleus after 30 min and 60 min of BMP-2 administration with similar levels like control cells, but did not display a strong initial peak as compared to control cells after 10 min of BMP-2 stimulation. In addition, the level of pSmad1/5/8 in the cytosolic fraction was lower relative to control cells (Figure 2C, lanes 9–16). These results indicate that inhibition of dynamin-dependent endocytosis affects Smad phosphorylation kinetics and concomitantly the dynamics of nuclear translocation.

After phosphorylation and nuclear translocation, Smads bind to specific motifs in promoter regions, recruit additional transcription factors and regulate transcription of target genes [7]. As already shown by us and by others, transcriptional activity is dependent on clathrin-mediated endocytosis [11]. To further investigate the effect of dynasore treatment on BMP-2 signaling, we examined its consequences on activation of the reportergene construct BRE-Luc after long and short term stimulation with BMP-2 [15]. As expected, the transcriptional activity was inhibited by dynasore after 6 h and 24 h of BMP-2-stimulation (Figure 2D,E).

### BMP-2 induced gene expression is differentially affected by inhibition of dynamin-dependent endocytosis

**Figure 3:**
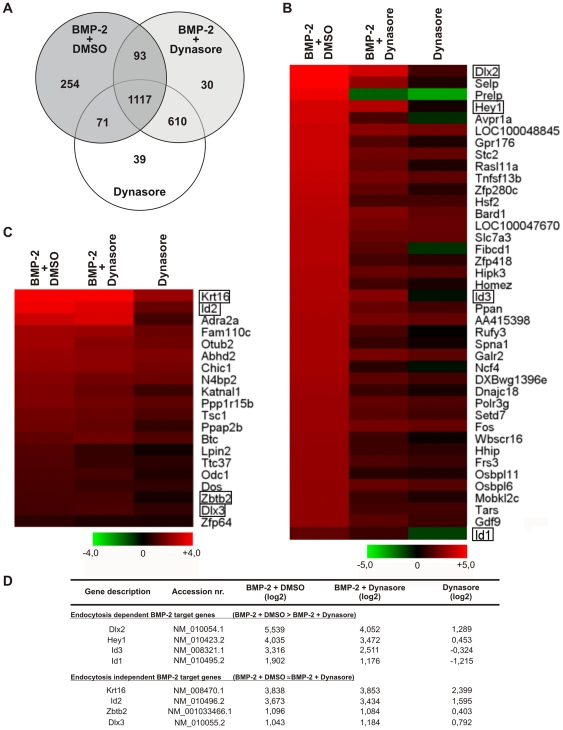
Gene expression is differentially affected by inhibition of dynamin-dependent endocytosis. Serum-starved C2C12 cells were treated for 2 h with 40 µM dynasore or 0.05% DSMO prior to stimulation with 30 nM BMP-2 for 6 h in medium containing dynasore or DMSO. RNA was isolated and subjected to whole genome profiling using the Illumina BeadChip system. Expression profiles were analyzed relative to DMSO control treatment. (**A**) Venn diagram based on significantly detected genes (1.4 fold regulation, detection P-value<0.05) (the entire gene list is depicted in Table S2). (**B**) Heatmap depicting a subset of BMP-2-induced genes, which are endocytosis-dependent (the complete list is given in Table S3). (**C**) Heatmap depicting BMP-2-induced genes which are endocytosis-independent (the gene list is shown in Table S4). (**B,C**) The heatmaps are colored by log_2_ expression signals according to the color key at the bottom. (**D**) Selected genes from lists representing endocytosis-dependent or -independent genes displayed in Table S3 and S4, respectively.

As shown above, phosphorylation of Smad1/5/8 and its nuclear translocation are retarded and diminished when endocytosis is blocked. The altered signaling kinetics resulted in an inhibitory effect of dynasore on BMP-2 induced BRE-Luc activity (Figure 2D,E). These results suggest that the accurate timing and signal intensity of the initial steps in BMP signaling is important for the transcriptional activation of target genes and that the initial steps are reliant on endocytosis. To further investigate the dependence of BMP-2 induced transcriptional activity on dynamin-dependent endocytosis, we performed a whole genome gene expression profiling using the Illumina BeadChip system. Serum-starved C2C12 cells were pre-treated for 2 h with dynasore, followed by BMP-2 stimulation for 6 h. Isolated RNA was subjected to microarray analysis. Expression profiles were normalized and genes that were significantly detected in all the treatments and either up- or down-regulated by 1.4 fold relative to DMSO control treatment were considered for further analysis (detection P-value<0.05). This list consists of 2214 genes that were commonly or exclusively regulated. Distinct and overlapping gene regulations are depicted in a Venn diagram (Figure 3A; the entire gene list is depicted in Table S2).

We identified genes that were exclusively regulated by BMP-2 in medium supplemented with vehicle (DMSO) only, (BMP-2+DMSO; 254 genes), by BMP-2 in the presence of dynasore (BMP-2+Dynasore; 30 genes) or by dynasore alone (Dynasore) (39 genes). We also identified genes that were commonly regulated by BMP-2+DMSO or Dynasore (93 genes), by BMP-2+DMSO or by Dynasore (71 genes) or by BMP-2+Dynasore or by Dynasore (610 genes). 1117 genes showed a common regulation in all three treatments.

In total, 925 genes were up-regulated by BMP-2+DMSO. These gene expression profiles were analyzed and those genes that showed nonspecific regulation by dynasore were not considered for further evaluation. Interestingly, we discovered genes that exhibited different expression patterns when the treatment BMP-2+DMSO was compared to the treatment BMP-2+Dynasore. In this way we identified two classes of genes that were differentially affected by inhibition of dynamin-dependent endocytosis (Figure 3B,C). The first class included 483 genes that showed reduced BMP-2-induced gene expression following treatment with dynasore (BMP-2+Dynasore) as compared to BMP-2+DMSO (Figure 3B; the complete gene list is depicted in Table S3). We defined this class of genes as endocytosis-dependent, since their expression levels were reduced following treatment with dynasore. Among those, we found known BMP-2 target genes like *Id1*, *Id3*, *Dlx2* and *Hey1* (Figure 3D). The second class included 20 genes whose expression levels were affected similarly following treatment with BMP-2+DMSO and in the presence of dynasore (BMP-2+Dynasore) (Figure 3C; the complete gene list is given in Table S4). We defined this class of genes as endocytosis-independent, since dynasore treatment did not affect their BMP-2 induced expression. Interestingly, this group also included known BMP-2 target genes such as *Id2* and *Dlx3*, as well as the genes *Krt16* and *Zbtb2* (Figure 3D).

### Confirmation of BMP-2 induced genes that are differentially affected by dynamin-dependent endocytosis

**Figure 4:**
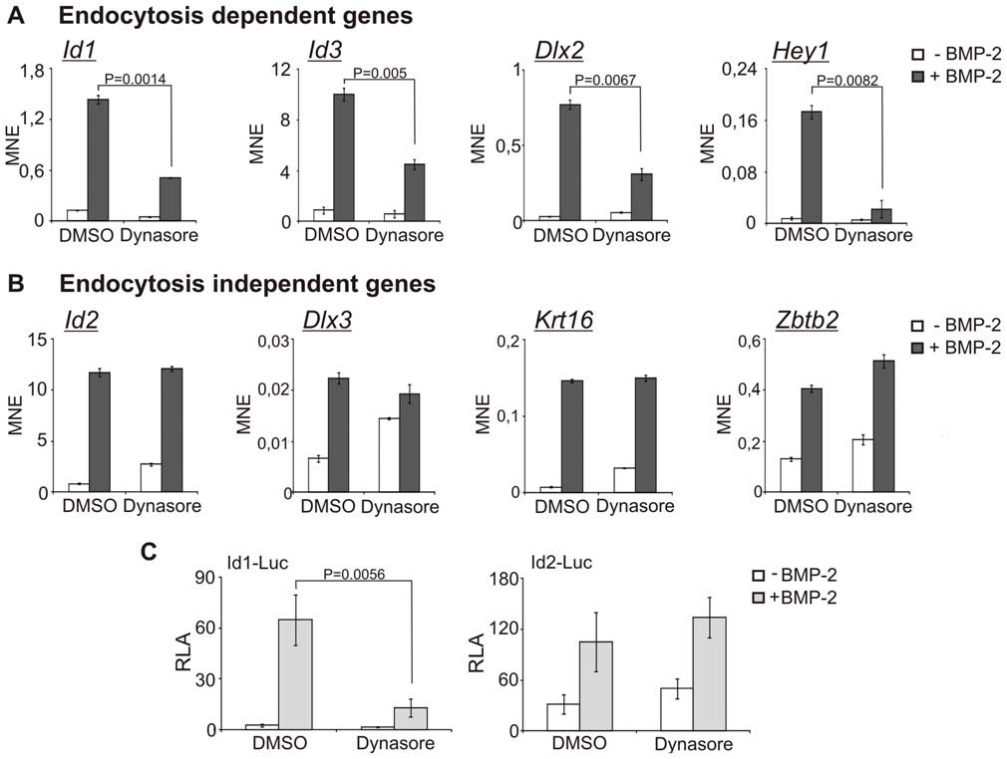
Validation of two classes of genes, which are dependent or independent of endocytosis. (**A,B**) Serum-starved C2C12 cells were treated for 2 h with 40 µM dynasore or 0.05% DMSO prior to stimulation with 30 nM BMP-2 for 6 h in medium containing dynasore or DMSO. Total RNA was isolated, cDNA was synthesized and mRNA expression was analyzed by qPCR using specific mouse primers (Table S1). Histograms show mean normalized expression (MNE) with standard error of duplicate measurements relative to the housekeeping gene *HPRT*. The analysis shown is representative of three independent experiments. (**C**) C2C12 cells were co-transfected with Id1-Luc or Id2-Luc and RL-TK. Cells were serum-starved and treated with 40 µM dynasore or 0.05% DMSO for 1 h prior to stimulation with 3 nM BMP-2 for 24 h in medium containing dynasore or DMSO. Relative luciferase activity (RLA) of Id1- or Id2-driven luciferase compared to constitutive expression of RL-TK is shown. Results are shown as mean ± s.d. of triplicate measurements, representative of three independent experiments.

In the microarray analysis we identified genes that are differentially affected by inhibition of dynamin-dependent endocytosis. To validate these findings, we performed quantitative real-time PCR (qPCR) analysis with independent samples. Cells were pre-treated with dynasore for 2 h and were subsequently stimulated with BMP-2 for 6 h. These experiments confirmed the existence of two classes of genes, whose expression in response to BMP-2 is either dependent or independent of endocytosis (Figure 4A,B). Expression of *Id1*, *Id3*, *Dlx2* and *Hey1* was endocytosis-dependent, as shown by the dynasore-mediated inhibition of their BMP-2 induced expression (Figure 4A). On the other hand, expression of *Id2*, *Dlx3*, *Krt16* and *Zbtb2* was endocytosis-independent (*i.e.*, no effect of dynasore) (Figure 4B). *Id1*, *Id2* and *Id3* (inhibitor of differentiation), which belong to the group of helix-loop-helix (HLH) transcription factors, are direct target genes of BMP signaling [16]. Interestingly, only *Id1* and *Id3* were inhibited by dynasore (Figure 4A), whereas *Id2* was not affected (Figure 4B). The difference between *Id1* and *Id2* gene expression in response to BMP-2 stimulation under endocytosis inhibition conditions was also confirmed in reportergene assays with reporter constructs containing the *Id1* or *Id2* promoter regions (Figure 4C) [17], [18]. Furthermore, *Dlx2* and *Dlx3* belong to the family of Distal-less homeobox genes and are important players in organ development [19]. Both proteins are known early target genes of BMP signaling in C2C12 cells [20]. Similar to the Id-family of proteins, *Dlx2* and *Dlx3* are differently affected upon inhibition of endocytosis, although they belong to the same protein family. Thus, BMP-2-stimulated *Dlx2* gene expression is inhibited by dynasore, whereas *Dlx3* is unaffected (Figure 4A,B). *Hey1* belongs to the hairy/Enhancer of split-related repressor protein basic helix-loop-helix family and is a direct BMP target gene [21]. *Hey1* expression upon BMP-2 addition is also affected by inhibition of dynamin-dependent endocytosis, resembling *Id1*, *Id3* and *Dlx2* (Figure 4A). In contrast, *Krt16* and *Zbtb2* expression was not affected by dynasore treatment during BMP-2 stimulation. Both proteins have not been described yet to be target genes in BMP signaling. Krt16 belongs to the type I keratin family of proteins and was shown to be involved in keratinocyte migration [22]. The zinc finger and BTB domain-containing protein 2 (Zbtb2) is described as a transcription factor that represses p53 function [23]. In addition to the microarray data, these results further support the notion that two classes of BMP target genes exist, which are differentially affected by inhibition of dynamin-dependent endocytosis and thus by altered Smad1/5/8 phosphorylation kinetics.

### Osteoblast differentiation of C2C12 cells is arrested by short-term inhibition of dynamin-dependent endocytosis

**Figure 5:**
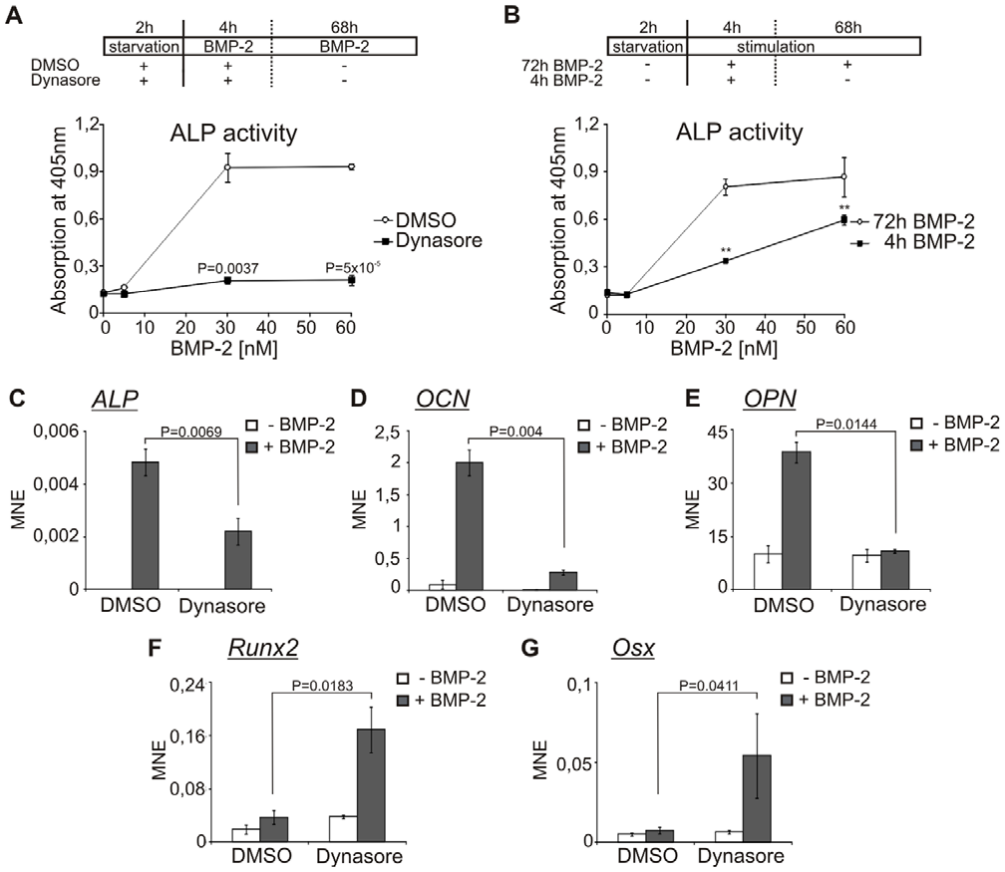
Osteoblast differentiation is arrested by inhibition of dynamin-dependent endocytosis. (**A,B**) Confluent C2C12 cells were serum-starved for 2 h prior to stimulation with the indicated concentrations of BMP-2. After 72 h of stimulation, cells were lysed and ALP activity was measured at 405 nm by conversion of para-nitrophenylphosphate. A schematic representation of the treatment is depicted above the respective histogram. The histograms show mean ± s.d. of triplicate measurements representative of three independent experiments. (**A**) During starvation and initial 4 h of stimulation, 40 µM dynasore or 0.05% DSMO were added. (P-values in relation to control treated samples). (**B**) After 4 h of stimulation, BMP-2-containing medium was replaced by medium without BMP-2. (** P-values in relation to unstimulated samples; 30 nM: P = 0.008, 60 nM: P = 0.0017) (**C–G**) Confluent C2C12 cells were treated as described in (A) with 30 nM BMP-2. Total RNA was isolated, cDNA was synthesized and mRNA expression was analyzed by qPCR using specific mouse primers (Table S1). Histograms show mean normalized expression (MNE) with standard error of duplicate measurements relative to the housekeeping gene *GAPDH*. The results are representative of three independent experiments.

BMPs are potent inducers of mesenchymal cells to differentiate into osteoblasts and chondroblasts [5]. The C2C12 precursor cell line provides a biologically suitable model to investigate the BMP-2-induced transcriptional cascade which initiates osteoblast differentiation [24]. We have previously shown that ALP production is dependent on Smad and non-Smad signaling, as well as on both clathrin- and caveolae-mediated endocytosis [6], [11]. Considering that inhibition of dynamin-dependent endocytosis affects Smad1/5/8 signaling kinetics and BMP-2-induced transcriptional expression profiles, we examined the consequences of these altered signaling properties on osteoblast differentiation. Differentiation was analyzed after 72 h of BMP-2 administration to serum-starved C2C12 cells, since the expression of osteoblast markers such as *alkaline phosphatase* (*ALP*), *osteocalcin* (*OCN*) and *osteopontin* (*OPN*) is detectable after this time period [24]. Application of dynasore for 2 h efficiently blocked endocytic uptake in C2C12 cells in our study and this effect was reversed by wash-out of the inhibitor [12]. However, long-term treatment over several days has lead to cell death. Therefore, dynasore was added only during starvation and the initial 4 h of BMP-2 stimulation in the differentiation assays. To test whether inhibition of endocytosis during the initial phase of BMP-2 signal transduction affects osteoblast differentiation, ALP assays were performed (Figure 5A). Application of dynasore during the first 4 h of BMP-2 stimulation significantly reduced ALP activity, even though the ligand was continuously present during the following 68 h. Application of the ligand for these additional 68 h did not circumvent the impaired ALP activity. The importance of the initial phase of stimulation was also demonstrated by ALP assays after 72 h, where BMP-2 was applied only during the initial 4 h (Figure 5B). These results suggest that correct signaling properties in the initial phase of BMP-2 stimulation are important for commitment of the cells towards the osteoblast lineage.

To gain more insight into the effect of inhibition of dynamin-dependent endocytosis in osteoblast differentiation, we performed qPCR analysis for osteoblast markers. Cells were pre-treated with dynasore and subsequently stimulated with BMP-2 for 72 h. During the initial 4 h of BMP-2 stimulation, dynasore was added. The expression analysis of osteoblast markers like *ALP*, *OCN* and *OPN* revealed that dynasore treatment results in down-regulation of these genes (Figure 5C–E). Interestingly, combining BMP-2 with dynasore resulted in higher and sustained expression levels of crucial early markers for osteoblast differentiation like *runt related transcription factor 2* (*Runx2*) and *osterix* (*Osx*) relative to control cells (Figure 5F,G). Taken together, the blockade of dynamin-dependent endocytosis during the initial phase of BMP-2 application resulted in down-regulation of osteoblast markers, thereby attenuating osteoblast differentiation. In contrast, the expression levels of early markers such as *Runx2* and *Osx* were increased by dynasore treatment.

## Discussion

Fine-tuning of signaling events is essential for living cells to regulate proper signal conversion and outcome. Endocytosis and recycling of membrane-associated proteins, including (but not limited to) receptor complexes and other signaling proteins, provides an important mechanism that regulates signal transduction. Moreover, endosomes derived from the plasma membrane can serve as signaling platforms for a number of signal transduction cascades, and certain signaling components are exclusively localized to endosomes [10]. The role of endocytosis in TGFβ signaling has been extensively investigated [25], whereas endocytic regulation of the BMP signaling pathway is less studied. We have previously shown that Smad dependent signaling is initiated *via* PFCs at the plasma membrane and specific transcriptional activation is mediated through clathrin-dependent endocytosis [11].

In this study, we show that dynamin-dependent endocytosis is a prerequisite for a functional BMP-2 induced transcriptional cascade to induce the majority of BMP/Smad target genes. We demonstrate that expression of those genes is differentially affected by inhibition of endocytosis, leading to attenuation of osteoblast differentiation of C2C12 cells.

We took advantage of the dynamin-dependent endocytosis inhibitor dynasore, which specifically interferes with the GTPase activity of dynamin and thereby inhibits pinching off of vesicles from the membrane, inhibiting both clathrin- and caveolae-mediated endocytosis [12].

Previous results from our group already demonstrated that Smad1/5/8 phosphorylation is induced at the plasma membrane as it was not affected by different endocytosis inhibitors when a single time point of 30 min BMP-2 stimulation was analyzed. Progression of the Smad dependent pathway by analysis of the transcriptional activation of the BRE-Luc reportergene was shown to be dependent on clathrin-mediated endocytosis [11]. Here, we expanded these studies using time-dependent assays of Smad phosphorylation. These studies confirmed that phosphorylation of Smad1/5/8 is still induced by BMP-2 when dynamin-dependent endocytosis is inhibited. However, new results from these studies are that dynasore treatment delays the dynamic profile and reduces the levels of pSmad1/5/8 formation (Figure 1C). Moreover, we show that the nuclear translocation dynamics of phosphorylated Smad1/5/8 are disturbed; the process was delayed, and the initial peak after 10 min of BMP-2 stimulation was missing (Figure 2A–C). In line with this finding, we demonstrated a down-regulation of BMP-2 induced activation of BRE-Luc activity in the presence of dynasore (Figure 2D,E).

In TGFβ signaling it was reported that Smad2 phosphorylation is a prerequisite for its detaching from the receptor and subsequent transcriptional activity [26], [27]. Our results suggest that efficient phosphorylation of Smad1/5/8 and downstream signaling is dependent on correct dynamics of vesicle formation and endocytosis. Considering that dynasore inhibits detachment of vesicles but leads to an accumulation of vesicle intermediates which are still connected to the plasma membrane [12], a disturbance of the localization of the receptors and other proteins involved in BMP signal transduction by interference with the endocytic machinery might contribute to the delay and reduction in Smad1/5/8 phosphorylation. However, a direct connection between endocytosis inhibition and altered Smad1/5/8 nuclear translocation dynamics after short treatment with BMP-2 cannot be stated at that point as delayed nuclear translocation might be a consequence of the delayed Smad1/5/8 phosphorylation profile and is not directly linked to altered endocytic processes in the cell. Further experiments on BMP-2 induced Smad1/5/8-BMPRI dissociation in the background of endocytosis inhibition will give more insights in that topic.

The BRE-Luc reportergene construct used in this study is derived from the *Id1* promoter but an artificial construct which was created to efficiently read out Smad1/5/8 dependent signaling [15]. As it does not display the endogenous full length *Id1* promoter, we were interested in the effect of endocytosis inhibition on endogenous BMP-2 induced genes.

To analyze global effects of BMP-2 stimulation under endocytosis inhibition conditions, we performed whole genome expression profiling of C2C12 cells. Our data showed 2214 genes that were significantly detected (detection P-value<0.05) and regulated by a cut-off of 1.4 fold (Figure 3A). 39 genes showed exclusive regulation by Dynasore. Among those, genes like *cell division cycle 27* (*Cdc27*) and *cyclin G1* (*Ccng1*) have been shown to be involved in cell cycle regulation [28], [29]. Regulation of these genes may be due to the known reversible effects of dynasore on cell growth [12]. 610 genes were commonly regulated by BMP-2+Dynasore or Dynasore. Among those, genes annotated to pathways such as oxidative phosphorylation, or involved in prostate cancer were regulated. Interestingly, we found genes like *Ephrin B1* (*Efnb1*) or *CAAT/enhancer binding protein beta* (*Cepbp*), which are known to be involved in cell migration, transcriptional regulation and control of osteogenic processes [30], [31].

We found 925 genes induced by BMP-2+DMSO. Those genes were differently affected by addition of dynasore. In this group we identified two classes of genes, which we defined as target genes dependent or independent of endocytosis (Figure 3B,C). The endocytosis dependence of genes in the first class was evident from the negative effect of dynasore on their expression (expression in the BMP-2+Dynasore treatment as compared to BMP-2+DMSO). Among those, we identified the known BMP-2 target genes *Id1*, *Id3*, *Dlx2* and *Hey1*, and genes that could be functionally annotated to Wnt signaling, pathways in cancer or cytokine-cytokine receptor interaction and that have critical roles in osteogenesis. For instance, *mitogen-activated protein kinase kinase kinase 7* (*Map3k7*) was shown to be upstream of p38 and its activation results in promoting Runx2 transcriptional activity and osteoblast differentiation [32]. *Interleukin 11* (*Il11*) was shown to positively influence bone formation and is suggested to enhance BMP action in bone [33]. The second class displayed genes that are independent of endocytosis, as judged by the finding that their expression was unaffected by dynasore addition during BMP-2 stimulation. Among those genes, we identified the BMP-2 target genes *Id2* and *Dlx3*, as well as the genes *Krt16* and *Zbtb2*. In addition, we detected the *transcription factor zinc finger protein 64* (*Zfp64*), which was described to be downstream of Runx2 and to be involved in osteogenic differentiation [34]. Moreover, we detected among the endocytosis-independent genes the EGF-like ligand *betacellulin* (*Btc*), which influences osteogenesis in MSCs [35].

To our knowledge, this is the first report, which shows that BMP-2 induced gene expression is selectively regulated by dynamin-dependent endocytosis; this was achieved by identifying genes whose expression is dependent or independent on endocytic signal transduction. Using qPCR studies, we confirmed those two gene classes, and demonstrated that BMP-2 induced *Id1*, *Id3*, *Dlx2* and *Hey1* gene expression is endocytosis-dependent, whereas *Id2*, *Dlx3*, *Zbtb2* and *Krt16* gene expression is endocytosis-independent (Figure 4A,B). All those genes, except *Krt16* and *Zbtb2*, were identified to be early target genes of BMP signaling and are associated with osteogenic processes [20], [21]. *Hey1* was shown to be a late early target gene of BMP-2 and knockdown of Hey1 in MSCs resulted in inhibition of osteoblast differentiation [21], [36].

**Figure 6:**
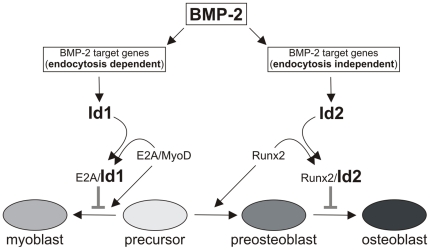
Hypothetical model. Id1 prevents myogenic differentiation by interfering with the transcriptional activity of E2A/MyoD complexes [16]. During early stages of osteoblast differentiation, *Runx2* is expressed. Expression levels of Runx2 need to be tightly controlled during later stages of differentiation, as Runx2 overexpression prevents late osteoblast differentiation [52]. In addition, it was demonstrated that Id2 prevents Runx2 binding to the late osteoblast marker promoter of *OCN* [38]. We suggest that differential gene expression regulation reliant on endocytic signal transduction provides an additional mechanism for the cell to regulate differentiation processes.

Id proteins are antagonists of bHLH transcription factors and are involved in transcriptional regulation. Id1 and Id3 show similar expression patterns during development and double knockout mice die during embryogenesis. Id1 associates with E2A proteins and interferes with the formation of a functional E2A-MyoD heterodimer, negatively influencing myogenesis [16], [37]. In contrast, Luan and colleagues reported that Id2 physically interacts with Runx2, inhibiting its osteogenic differentiation potential by preventing binding of Runx2 to the *OCN* promoter [38]. Those studies underline the opposing roles of Id1 and Id2 in differentiation. Interestingly, we could show that *Id1* expression was dependent on endocytosis whereas *Id2* expression was not (Figure 3, 4A,B). The differential regulation of *Id1* and *Id2* gene expression was also demonstrated in reportergene assays (Figure 4C). These results suggest that the mode and frequency of endocytosis in C2C12 cells might alter their direction of differentiation (osteogenesis *versus* myogenesis) by inducing *Id1 versus Id2* (Figure 6). Both pathways depend on phosphorylated Smad1/5/8; however, the kinetics and site of release from the receptor (endosome or plasma membrane) may determine the fate of activated Smads for further interactions with co-activators or co-repressors, leading to specific recognition of promoter sequences.

Interestingly, *Dlx2* and *Dlx3* are differentially regulated by dynasore treatment, although both belong to the same family of Distal-less homeobox proteins (Figure 3, 4A,B). SiRNA-mediated knockdown of Dlx3 resulted in down-regulation of *Runx2* gene expression, highlighting a role for Dlx3 in early stages of osteoblast differentiation [39]. In addition, recent publications reported that miR-31 targets both *Dlx3* and *Krt16* along with Fgf10, Sclerostin and BAMBI in hair follicles [22], [40]. These data underline the similarity in gene expression regulation of *Dlx3* and *Krt16* as both are identified in the small class of BMP target genes, which are independent of dynamin-controlled endocytosis (Figure 3, 4B).

The impact of endocytosis on transcription of target genes has been previously demonstrated for insulin signaling. Expression of the target genes *glucokinase* or *c-fos* is specifically initiated by insulin receptor isoform B (IR-B) located at the plasma membrane or in sorting endosomes, respectively [41]. Interestingly *c-fos* is also regulated by BMP-2 *via* the endocytosis-dependent Smad pathway (Figure 3B).

Endocytosis-independent Smad signaling was shown for activin stimulation, where an activin type I receptor mutant impaired in endocytic uptake was still able to induce Smad2 phosphorylation and subsequent transcriptional activation [42]. Activation of the TGFβ mediated Smad2/3 pathway was proposed to include presentation of Smads *via* Smad anchor for receptor activation (SARA), clathrin-mediated endocytosis of activated receptors and release from the receptors in early endosomes [9]. However, there are controversial reports on the necessity of clathrin-mediated endocytosis of phosphorylated Smad2 to induce transcription [43], [44]. Another endosomal protein, endofin, was found to positively influence TGFβ signaling [45], [46].

The association of the TGFβ and BMP receptors with specific adaptor proteins for clathrin-mediated endocytosis might be important to trigger this step in time resolved signal transduction. For the BMP receptor type II (BMPRII), interaction with clathrin adaptor proteins Eps15R and AP2 has been demonstrated [11], [47]. Spatial segregation of Smad signaling as shown here by altered phosphorylation dynamics might be triggered through this initial step of adaptor protein interaction allowing or excluding the receptors to internalize *via* dynamin dependent endocytosis events. However, because dynasore imparts a general blockade of endocytosis, it is possible that the inhibition of signaling by dynasore is due to a requirement for endocytosis or specific intracellular localization of another downstream component in the pathway. In order to resolve both segregated Smad pathways in more detail, specific Smad-interactome studies by concomitant BMP-2 stimulation and endocytosis inhibition would give more insights into the selective regulation.

The physiological importance of our findings is highlighted in osteoblastic differentiation studies. We show that dynasore treatment during the initial 4 h of BMP-2 induced differentiation inhibited ALP activity, which goes hand in hand with the down-regulation of the osteoblast marker genes *ALP*, *OCN* and *OPN* in qPCR analysis (Figure 5A, C–E). Interestingly, the crucial early marker genes *Runx2* and *Osx* [48], [49] were up-regulated (Figure 5F,G). The importance of concise Runx2 expression levels at multiple stages during differentiation processes has been extensively reviewed [50], [51]. In addition to this, Liu and colleagues reported that over-expression of Runx2 inhibited late stages of osteoblast differentiation [52]. The requirement of a confined, spatiotemporal expression pattern as described for Runx2 could also be demonstrated for Osx. The forced expression of Osx in different cell types was incapable of inducing osteoblast differentiation [53], [54]. Our observations suggest that interference with the BMP-2-induced transcriptional cascade by dynasore might lead to the entrapment of cells in an early osteoblast stage, which cannot progress towards late osteoblast differentiation due to disturbed expression of important early osteoblast markers Runx2 and Osx. Our results indicate that negative Runx2 regulators are induced *via* the endocytosis-dependent BMP-2 signaling pathway. The transcription factor E4BP4 and the homeodomain protein Nkx3.2 were already described as negative regulators of Runx2 expression [55], [56]. Id2 inhibits Runx2 activity by physical interaction, which prevents Runx2 binding to the *OCN* promoter [38]. Since we analyzed gene expression on a whole-genome level after 6 h of BMP-2 stimulation, and Runx2 down-regulation is needed during late stages of osteoblast differentiation (24 h [51]), further analysis during the complete differentiation process will help to understand the underlying mechanism and to identify all crucial players.

The importance of the initial stimulation phase in BMP-induced differentiation was described by van Bezooijen and colleagues, who reported that BMPs need to be present for 24 h on pre-osteoblast KS483 cells to induce osteoblast differentiation [57]. In line with this notion, we demonstrated that application of BMP-2 for a minimum of 4 h is sufficient to induce osteoblast differentiation in C2C12 cells (Figure 5B).

In summary, we show here for the first time that BMP/Smad signaling is spatially segregated into the predominant dynamin-dependent endocytosis path and a second pathway, which is endocytosis-independent. These pathways differ in their kinetics and in the regulation of distinct target genes, thus allowing for differential regulation of members within the Id and Dlx family. Functional interference with the dynamin-dependent pathway in the initial phase of osteoblastic differentiation arrests precursor cells, suggesting that the early stages of differentiation are strongly dependent on this route of the Smad pathway. Furthermore, we emphasize that treatment with BMP-2 for a limited time is sufficient to promote the osteoblast differentiation program.

## Materials and Methods

### Cell culture, transfection and materials

C2C12 mouse mesenchymal precursor cells (ATCC) were grown at 37°C with 10% CO_2_ in Dulbecco's Modified Eagle Medium (DMEM) (Biochrom) supplemented with 10% (v/v) Fetal Calf Serum (FCS), 1 mM Glutamine, Penicillin (100 units/ml) and Streptomycin (10 µg/ml) (PAA). Cells were transfected using Lipofectamine 2000 (Invitrogen) according to manufacturer's instructions and assayed 24 h after transfection. The ligand BMP-2 was generously provided by W. Sebald (University of Wuerzburg, Wuerzburg, Germany). Human transferrin labelled with AlexaFluor594 (Invitrogen) was stored light protected at 4°C as a stock solution of 5 mg/ml containing 2 mM sodium azide according to manufacturer's instructions. The endocytosis inhibitor dynasore (Sigma) was aliquoted under argon as a stock solution of 80 mM in DMSO and kept light protected at −80°C [58]. The following antibodies were used: anti-pSmad1/5/8 (#9511, Cell Signaling), anti-GAPDH (#2118, Cell Signaling), anti-Smad1 (sc-7965, Santa Cruz Biotechnology) and anti-Histon H3 (#9715, Cell Signaling).

### Inhibition of dynamin-dependent endocytosis

To inhibit endocytosis in signaling assays, serum starved C2C12 cells were washed with PBS and pre-treated with 40 µM dynasore or 0,05% DMSO as control in DMEM supplemented with 0% FCS, 1 mM Glutamine, Penicillin (100 units/ml) and Streptomycin (10 µg/ml) (serum free medium) for 30 min up to 2 h. During stimulation treatments, dynasore and DMSO were added to the indicated medium if not stated otherwise.

### Transferrin uptake

To assess endocytosis inhibition efficiency of dynasore, C2C12 cells were subjected to transferrin uptake [14]. Cells were grown on glass coverslips and pre-treated with 40 µM dynasore or 0,05% DMSO in serum free medium in parallel with the respective experiment. The uptake experiment was performed by incubating the cells in 30 µg/ml Alexa594-transferrin in Opti-MEM (Invitrogen) including dynasore or DMSO for 15 min at 37°C. Cells were put on 4°C and washed 3 times with ice-cold PBS/10 mM MgCl_2_ before fixation with 4% PFA for 20 min at room temperature. Cells were washed two times with PBS and incubated for 3 min at −20°C with methanol. After 5 washes with PBS, cells were stained with 1 µg/ml Hoechst dye in PBS for 2 min at room temperature and mounted in FluoromountG (Southern Biotech). Cells were imaged with 63-fold magnification with an Axiovert 200 M fluorescence microscope (Zeiss) by using the same exposure time for all slides. Pictures were analyzed using SlideBook Software (Intelligent Imaging Innovations, Inc.) by quantifying the total amount of Alexa594-transferrin accumulated within cell boundaries. Transferrin uptake is represented in arbitrary units (AU) as mean ± s.d. of fluorescence intensity inside of a minimum of 400 cells for each condition.

### Smad phosphorylation assay and Cytosplasmic-Nuclear fractionation assay

C2C12 cells were starved for 22 h in DMEM supplemented with 0,5% FCS, 1 mM Glutamine, Penicillin (100 units/ml) and Streptomycin (10 µg/ml), then washed once with PBS and incubated with serum free medium including 40 µM dynasore or 0,05% DMSO for 2 h. Cells were subsequently stimulated with 10 nM BMP-2 in serum free medium containing dynasore or DMSO for the indicated time periods. To analyze the Smad phosphorylation status, cells were directly lysed in 1× Laemmli-buffer. To analyze the subcellular localization of Smad proteins by fractionation, cells were prepared according to manufacturer's instructions using ProteoJet Cytoplasmic and Nuclear Protein Extraction Kit (Fermentas). Samples were subjected to SDS-PAGE and transferred to nitrocellulose membranes by Western blotting. The membranes were probed with antibodies specific for C-terminal phosphorylated Smad1/5/8 and GAPDH or Histone H3 as loading control or control for fractionation, respectively.

### Immunofluorescence microscopy

To analyze subcellular localization of Smad1 protein by immunofluorescence, C2C12 cells were grown on glass coverslips and starved in serum free medium for 1,5 h. Cells were washed with PBS and pre-treated for 30 min in serum free medium containing 40 µM dynasore or 0,05% DMSO prior to stimulation with 10 nM BMP-2 for 30 min in serum free medium containing dynasore or DMSO. Subsequently, cells were fixed in 4% PFA for 20 min at room temperature and probed with anti-Smad1 antibody and an AlexaFluor594 goat anti mouse antibody (Invitrogen) [59]. Cells were stained with 1 µg/ml Hoechst dye in PBS for 2 min at room temperature and mounted in FluoromountG (Southern Biotech). Cells were analyzed with 63-fold magnification using an Axiovert 200 M fluorescence microscope. Quantification of relative fluorescence in the nucleus was assessed using SlideBook Software (Intelligent Imaging Innovations, Inc.) to determine the ratio of fluorescence intensity of Smad1 localization in the nucleus versus Smad1 localization in the cytoplasm of a minimum of 100 cells. The results are represented in arbitrary units (AU) as mean ± s.d.

### Reportergene Assay

To analyze BMP-2-dependent transcriptional responses, C2C12 cells were transfected with a BMP response element reportergene construct (BRE-Luc) [15], a reporter construct containing the promoter regions of Id1 ((−1575)-(+88), Id1-Luc) [17] or Id2 ((−4516)-(+80), Id2-Luc) [18] together with a constitutively active renilla luciferase (RL-TK) (Promega) as internal control. After 24 h, cells were starved for 3 h in serum free medium, washed with PBS and pre-treated for 1 h in serum free medium containing 40 µM dynasore or 0,05% DMSO prior to stimulation with 3 nM BMP-2 for 6 h or 24 h. During stimulation for 24 h, 0,5% FCS was added to the medium. Cell lysis and luciferase measurements were carried out according to manufacturer's instructions (Dual-Luciferase Reporter Assay System, Promega) and measured using a Mithras LB 940 luminometer (Berthold Detection Systems). The ratio of BMP-2 induced luciferase and renilla luciferase activity is shown as relative luciferase activity (RLA). The results are represented as mean ± s.d. of triplicates.

### Illumina BeadChip hybridization and data analysis

C2C12 cells were starved for 2 h in serum free medium, washed with PBS and pre-treated for 2 h in serum free medium containing 40 µM dynasore or 0,05% DMSO and then stimulated with 30 nM BMP-2 in serum free medium containing dynasore or DMSO for 6 h. Samples were subjected to gene expression analysis as biological duplicates.

Whole genome mRNA expression analysis was performed using MouseRef-8 v2.0 Expression BeadChips (BD-202-0202, Illumina). Total RNA was isolated using NucleoSpin RNA II Isolation Kit (Machery-Nagel) followed by quality assessment using both spectrophotometer (NanoDrop Technologies) and agarose gel electrophoresis. A total of 400 ng RNA was used as input for microarray experiments. Biotin-labelled cRNA was generated, employing a linear amplification kit (Ambion). Subsequently hybridization, washing, Cy3-streptavidin staining and scanning of hybridized chips was performed using Illumina Beadstation 500 platform (Illumina) according to manufacturer's instructions. For gene expression data analysis, raw data were normalized using the rank invariant normalization algorithm included in BeadStudio 3.0 software (Illumina). To assess correlation coefficients, BeadStudio 3.0 software was utilized (Figure S1). For obtaining the list of significant differentially regulated genes, one-way ANOVA was performed using TIGR-MEV software [60], [61]. A gene was considered to be significantly regulated, only if the corresponding P-values for both detection and significance were <0.05. Since the variation between the replicates of the treatment DMSO+BMP-2 was high (correlation coefficient of 0.8351), only one of the replicates was considered for further analysis. The excluded sample was not considered for the significance test, although the detection P-values and fold change were used for filtrations. A cut-off of >1.4 fold change with respect to DMSO control treatment was set to extract differentially expressed genes in group-wise comparisons. A Venn diagram was generated from these regulated lists employing the VENNY interactive tool (http://bioinfogp.cnb.csic.es/tools/venny/index.html) and heat maps were generated using the TIGR-MEV software [60], [61]. All microarray data presented in this study is MIAME compliant and has been submitted to GEO database (accession number GSE29373). Functional annotation and pathway analysis were done using the DAVID platform version 6.7 with default parameter settings [63], [64]. However, a subset of the regulated genes from each comparison was validated by quantitative real-time PCR (qPCR), to ascertain the reliability of the data. This was performed with cDNA generated from RNA used for whole genome analysis as well as with RNA from two independent experiments.

### qPCR analysis

To analyze mRNA expression after 6 h of BMP-2 stimulation (Figure 4A B), C2C12 cells were treated like described above in microarray analysis. To analyze the effect of inhibition of endocytosis by dynasore on mRNA expression after initiation of osteoblast differentiation (Figure 5C–G), cells were treated as described below for ALP assay. Total RNA was isolated according to manufacturer's instructions using NucleoSpin RNA II Isolation Kit (Machery-Nagel). Complementary DNA (cDNA) was synthesized from 0,5–1,5 µg RNA and used in SYBRGreen qPCR. The amount of investigated transcript was determined relative to the housekeeping genes *hypoxanthine-phosphoribosyltransferase* (*HPRT*) or *glycerinaldehyd-3-phosphat-dehydrogenase* (*GAPDH*). The sequences of mouse specific primers for qPCR analysis are shown in Table S1. All measurements were done in duplicates (StepOnePlus Real-Time PCR System, Applied Biosystems) and C(T) values were determined with StepOne Software (Applied Biosystems). Mean normalized expression (MNE) and the corresponding standard error were calculated according to a previous publication [62].

### Alkaline phosphatase assay

C2C12 cells were seeded at high density to reach confluency after 24 h. Cells were washed with PBS, starved for 2 h in serum free medium and subsequently stimulated with 5 nM, 30 nM and 60 nM BMP-2 in DMEM supplemented with 2% FCS, 1 mM Glutamine, Penicillin (100 units/ml) and Streptomycin (10 µg/ml) for 72 h. To study the effect of endocytosis inhibition on osteoblast differentiation by measuring ALP activity (Figure 5A), 40 µM dynasore or 0,05% DMSO were included during starvation and initial 4 h of stimulation with BMP-2 in serum free medium, then stimulation medium was replaced by medium containing BMP-2 only. To study the importance of the initial phase of BMP-2 stimulation on ALP activity (Figure 5B), BMP-2 in the indicated concentration was only added for 4 h and afterwards the cells were incubated in medium without BMP-2 for additional 68 h. Cells were lysed by incubation for 1 h in 100 µl ALP 1 buffer (0.1 M glycine pH 9.6, 1 mM MgCl_2_, 1 mM ZnCl_2_, 1% v/v NONIDET P-40). After addition of 100 µl ALP 2 buffer (0.1 M glycine pH 9.6,1 mM MgCl_2_,1 mM ZnCl_2_, 2 mg/ml para-nitrophenylphosphate (pNPP) (Roth)) ALP enzymatic activity was measured using a microplate reader (Tecan) at 405 nm. The results are represented as mean ± s.d. of triplicates.

### Statistical analysis

Results are presented as mean ± standard deviation (s.d.) or standard error (s.e.). Statistical significances (P-values) were calculated by two-tailed Student's *t* test. P-values of P<0.05 were considered to indicate statistical significance.

## Supporting Information

Figure S1 **Linear correlation coefficients of biological duplicates subjected to whole genome expression analysis.**
(TIF)

Table S1 **List of mouse primers used for qPCR analysis.**
(DOC)

Table S2 **Complete lists of genes regulated and significantly detected in microarray analysis (** **Figure 3A** **).** Worksheets: 1) genes exclusively regulated by BMP-2+DMSO (254 genes), 2) genes exclusively regulated by BMP-2+Dynasore (30 genes), 3) genes exclusively regulated by Dynasore (39 genes), 4) genes commonly regulated by BMP-2+DMSO or BMP-2+Dynasore (93 genes), 5) genes commonly regulated by BMP-2+DMSO or by Dynasore (71 genes), 6) genes commonly regulated by BMP-2+Dynasore or by Dynasore (610 genes), 7) genes commonly regulated in all treatments (1117 genes). Expression signals relative to DMSO are displayed in log_2_ scale, detection P-values of all treatments are included.
(XLS)

Table S3 **List of BMP-2 induced genes which are dependent on dynamin-dependent endocytosis (** **Figure 3B** **) (483 genes).** Expression signals relative to DMSO are displayed in log_2_ scale, detection P-values of all treatments are included.
(XLSX)

Table S4 **List of BMP-2 induced genes which are independent of dynamin-dependent endocytosis (** **Figure 3C** **) (20 genes).** Expression signals relative to DMSO are displayed in log_2_ scale, detection P-values of all treatments are included.
(XLSX)
