# Supplementary figures and images for: Spatial Segregation of BMP/Smad Signaling Affects Osteoblast Differentiation in C2C12 Cells
Source: PLoS One. 2011 Oct 5;6(10):e25163. doi: 10.1371/journal.pone.0025163 (PMC3187766; doi:10.1371/journal.pone.0025163)

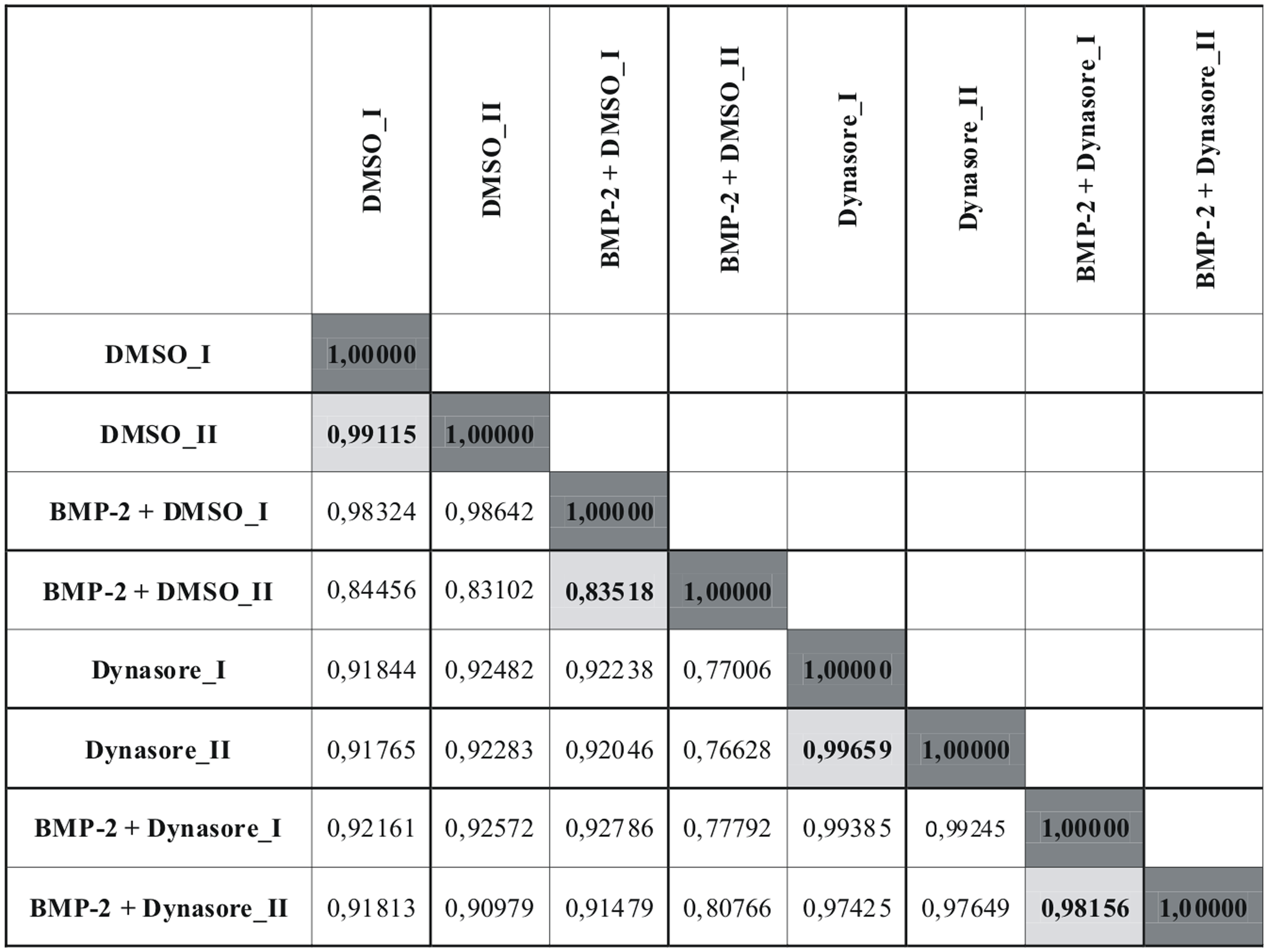

Supplement: Figure S1 — Linear correlation coefficients of biological duplicates subjected to whole genome expression analysis. (TIF) [file pone.0025163.s001.tif]
