# Supplementary material for: Spatial Segregation of BMP/Smad Signaling Affects Osteoblast Differentiation in C2C12 Cells
Source: PLoS One. 2011 Oct 5;6(10):e25163. doi: 10.1371/journal.pone.0025163 (PMC3187766; doi:10.1371/journal.pone.0025163)
Supplement: Table S1 — List of mouse primers used for qPCR analysis. (DOC) [file pone.0025163.s002.doc]

**Table S2. List of mouse primers used for qPCR analysis.**

| **Gene name** | **forward primer** | **reverse primer** |
| --- | --- | --- |
| **Alkaline phosphatase (ALP)** | 5’-AACCCAGACACAAGCATTCC-3’ | 3’-GAGAGCGAAGGGTCAGTCAG-5 |
| **Distal-less homeobox 2 (Dlx2)** | 5’-AACCACGCACCATCTACTCC-3’ | 3’-CGCTTTTCCACATCTTCTTGA-5’ |
| **Distal-less homeobox 3 (Dlx3)** | 5’-AGTATCTGGCCTTGCCTGAG-3’ | 3’-ACTGTTGTTGGGGCTGTGTT-5’ |
| **Glycerinaldehyd-3-phosphat-dehydrogenase (GAPDH)** | 5’-TGCACCACCAACTGCTTAG-3’ | 3’-GAGGCAGGGATGATGTTC-5’ |
| **Hairy/enhancer-of-split related with YRBW motif 1(Hey1)** | 5’-TGGATCACCTGAAAATGCTG-3’ | 3’-ATGCTCAGATAACGGGCAAC-5’ |
| **Hypoxanthine-phosphoribosyltransferase (HPRT)** | 5’-TGTTGTTGGATATGCCCTTG-3’ | 3’-ACTGGCAACATCAACAGGACT-5’ |
| **Inhibitor of differentiation 1 (Id1)** | 5’-CTTCAGGAGGCAAGAGGAAA-3’ | 3’-CAAACCCTCTACCCACTGGA-5’ |
| **Inhibitor of differentiation 2 (Id2)** | 5’-GGACATCAGCATCCTGTCCTT-3’ | 3’-TCTCCTGGTGAAATGGCTGA-5’ |
| **Inhibitor of differentiation 3 (Id3)** | 5’-AGCTCACTCCGGAACTTGTG-3’ | 3’-TGAAGAGGGCTGGGTTAAGA-5’ |
| **Keratin 16 (Krt16)** | 5’-GCAGATGGCAGAGAAGAACC-3’ | 3’-GCGGTTGCTCTGGATTAGAT-5’ |
| **Osteocalcin (OCN)** | 5’-GCAGGAGGGCAATAAGGTAG-3’ | 3’-CTTTAGGGCAGCACAGGTC-5’ |
| **Osteopontin (OPN)** | 5’-GCTTGGCTTATGGACTGAGG-3’ | 3’-GGGATGACATCGAGGGACT-5’ |
| **Osterix (Osx)** | 5’-CCCTTCTCAAGCACCAATGG-3’ | 3’-GATACGTTTACTGATGGGTGGGA-5’ |
| **Runt-related transcription factor 2 (Runx2)** (detects variant I and II) | 5’-TAAGAAGAGCCAGGCAGGTG-3’ | 3’-GGTTGGGTGCTTACGTGAT-5’ |
| **Zinc finger and BTB domain  containing 2 (Zbtb2)** | 5’-TTGGTTTCCTGTGTGACTGC-3’ | 3’-ACGCATTCACTGGTCTGATG-5’ |
